# UTI assessment tool for intermittent catheter users: a way to include user perspectives and enhance quality of UTI management

**DOI:** 10.1186/s12912-022-01033-7

**Published:** 2022-10-06

**Authors:** S. V. Lauridsen, M. A. Averbeck, A. Krassioukov, R. Vaabengaard, S. Athanasiadou

**Affiliations:** 1 Department of Urology, Copenhagen University Hospital, Copenhagen, Denmark; 2 WHO-CC, Parker Institute, Copenhagen University Hospital, Frederiksberg and Bispebjerg Hospital, Frederiksberg, Denmark; 3 Moinhos de Vento Hospital, Porto Alegre, Brazil; 4 International Collaboration on Repair Discoveries (ICORD), Division of Physical Medicine and Rehabilitation, Department of Medicine, Faculty of Medicine, International Collaboration on Repair Discoveries (ICORD), University of British Columbia, Endowment Lands, Canada; 5 G.F. Strong Rehabilitation Centre, Vancouver Coastal Health, Vancouver, British Columbia Canada; 6 Coloplast A/S, Humlebaek, Denmark

**Keywords:** Intermittent catheterisation, UTI, Urinary tract infection, User perspective, Neurogenic bladder, Assessment tool, Nursing

## Abstract

**Background:**

Urinary Tract Infections (UTIs) are among the most severe complications for users of intermittent catheterisation (IC), with numerous risk factors contributing to their occurrence. The aim of this study was to develop a tool to assess UTI risk factors among IC users in a systematic way that considers the perspective of the individual user.

**Methods:**

The Design Thinking Process was used to guide the development of the content and format of the tool. The UTI Risk Factors model by Kennelly et al. was used as a basis for developing the content. Insights on the appropriate content and format were collected via the Coloplast Nurse Advisory Boards and by conducting a qualitative evidence synthesis on user perspectives and practices in relation to UTIs.

**Results:**

The literature search identified a total of 3544 articles, out of which 22 met the inclusion criteria. Additionally, three rounds of meetings were conducted with approximately 90 nurses from the Nurse Advisory Boards across Europe. The qualitative evidence synthesis showed that users describe their UTI symptoms in different terms and that personal needs and priorities impact their adherence and catheter selection. Furthermore, some users lack relevant and updated knowledge about IC and UTIs.

The nurses described that correct UTI diagnosis is essential. They pointed that they would assess the user’s general condition, adherence, technique, and catheter type as potential areas of risk factors and emphasised the importance of adequate support for users.

The study resulted in the development of the UTI assessment tool for intermittent catheter users, which comprises three elements: a guide for healthcare professionals, a dialogue board, and a notepad. The tool starts with a confirmation of the UTI incidence, and then assesses risk factors via questions on health, adherence, technique, and catheter, and concludes with a support section.

**Conclusions:**

The UTI assessment tool for intermittent catheter users is designed to help healthcare professionals assess UTI risk factors in a systematic way, while engaging users and taking their perspective into account. By identifying the relevant risk factors, the use of this tool has the potential to reduce the occurrence of UTIs for the individual IC user.

## Background

Adult neurogenic lower urinary tract dysfunction (NLUTD) is defined as *“abnormal or difficult function of the bladder, urethra in mature individuals in the context of clinically confirmed relevant neurologic disorder”* [1]. Distinct dysfunctional patterns in the lower urinary tract may appear, depending mostly on the site and severity of the neurological lesion [2–4]. Urinary retention, that is an inability to properly empty the bladder, is a frequent finding in patients with suprasacral lesions, as in traumatic spinal cord injury (SCI) and multiple sclerosis (MS) [1]. Data on the prevalence and incidence of underlying neurological disorders, as well as the occurrence of secondary neuro-urological symptoms vary widely, reflecting the high variability in the neurological cohort included in studies. Annual crude incidence rates in traumatic SCI vary from 12,1 per million in the Netherlands to 57,8 per million in Portugal [5]. Multiple sclerosis (MS), which is the most common neuro-inflammatory disorder of the central nervous system affects 5 in 100.000 people in low-risk areas. The prevalence of NLUTD is high in MS patients with > 80% reporting storage and/or voiding symptoms and up to 91% presenting neurogenic detrusor overactivity [6].

Urinary tract infections (UTIs) are a common and potentially severe complication for people with NLUTD [7, 8]. Intermittent catheterisation (IC) has been established as the “gold standard” method of bladder management for the neurogenic population given its relatively lower risk of inducing UTIs and other severe complications, compared to, for example, indwelling catheterisation [9, 10]. However, even with IC, UTIs are a prevalent problem across regions [11]. The UTI incidence ranges between 0.8-3.5 events per year in the community or higher in hospitals and rehabilitation centres, while the assessment of UTIs can be difficult in neurological patients [8, 9, 12].

The severity and frequency of UTIs has driven extensive research into their causes, pathogenesis, prevalence, and treatment [8, 9, 12–16]. This has clearly illustrated that UTIs are a serious and multifactorial condition, and that a proactive approach is required to manage them at their causes [9, 17]. The UTI Risk Factors model published in 2019 aims to compile the risk factors with the potential to cause UTIs and include the evidence behind them [12]. The model shows that some risk factors are related to the user’s health condition and the catheter products used, while others are related to users’ behaviours. While there is extensive literature addressing health conditions and products, only a small proportion of the literature is devoted to the users’ behaviours and perspectives related to UTIs [18].

Users’ decisions are not always aligned with clinical evidence. Users struggling with recurrent UTIs are found to dropout or transition to higher-risk methods, such as indwelling catheters [19–22], which underlines the importance of understanding their needs and the impact IC and frequent UTIs have on their quality of life. At the same time, little is known about how health care professionals (HCPs) assess users with recurrent UTIs and how the users’ perspective is taken into consideration during a consultation [10]. This implies a need to understand both the users’ and the HCP’s perspectives concerning UTIs to raise the assessment standard for individual users.

Therefore, the purpose of this study has been to develop a tool that will help HCPs evaluate UTI risk factors in a systematic way that considers each user’s perspective, and identify areas requiring interventions.

## Methods

**Fig. 1:**
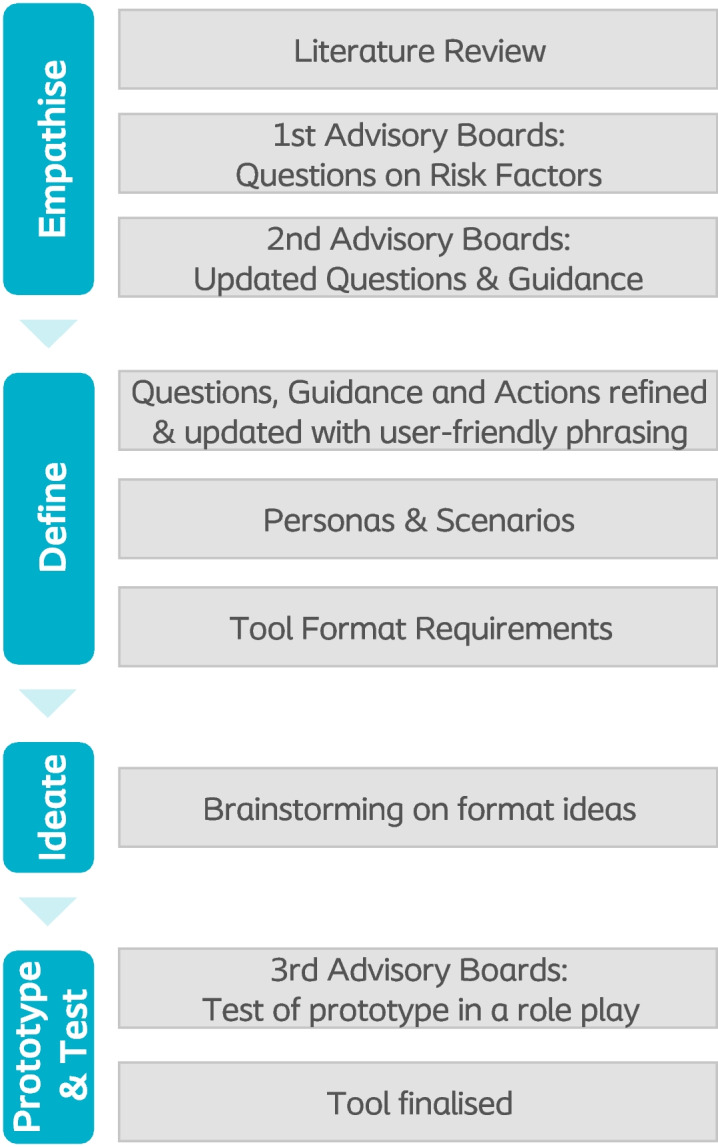
Process of developing the UTI assessment tool for intermittent catheter users

The development of this tool required a robust process to define the content and a suitable format relevant for the clinical practice. Therefore, the five steps of the Design Thinking Process (Empathise, Define, Ideate, Prototype and Test) [23] was used. This study required insights on current clinical practice, which were collected by combining two methodologies. We collected input from experienced nurses participating in the Coloplast Nurse Advisory Boards across Europe. Then we performed a qualitative evidence synthesis of intermittent catheters users experience with UTIs [24]. These insights were transformed into the UTI assessment tool for intermittent catheter users by following different methodologies, according to the Design Thinking Process. An overview of the methodologies is depicted in Fig. 1.

### Qualitative evidence synthesis

A literature search was conducted, focusing on the users’ perspective of UTIs and symptoms, as well as their behaviours and practices associated with UTI risk factors. The search was performed in PubMed, using the following strategy:

“Intervention” AND “Type of Study”, where:

- Intervention: Intermittent (Urethral) Catheter/Catheterisation OR Urinary Catheter/Catheterisation OR Clean Intermittent Catheter/Catheterisation OR (Urethral) Self-Catheterisation
- Type of Study: “Qualitative research” OR “Qualitative study”

The inclusion criteria were qualitative studies on user perspectives on IC, adults with neurogenic lower urinary tract dysfunction, IC as an emptying method and publications in the English language. The articles were first screened based on the title, abstract and keywords, and subsequently based on full text. Articles were excluded if they referred to diagnosis, surgery, treatment or other medical intervention, conditions, complications not related to UTIs, educational initiatives,.

Information related to experiences with UTIs, as well as practices and perceptions that could be related to the UTI Risk factors were identified. If applicable, the information was grouped under the related risk factors, or under new descriptive headers. These groups gave rise to themes encompassing the main literature findings.

### Nurse advisory board input

Consensus meetings with Nurse Advisory Boards were conducted to identify the risk factors relevant to assessing and define a way to uncover them. These well-established boards consist of nurses who work in urology and rehabilitation clinics and cover users with different diagnoses with NLUTD, bringing their expertise from hospital and community settings. Approximately 90 nurses from different European countries participated in meetings held in three rounds between January and December 2021. Not all nurses participate each time. We have estimated that approximately 30 participated in the first round, 20 in the second and 80 in the third.

In the first round, the nurses discussed which factors from the UTI Risk Factor model are relevant to assess in clinical practice [12]. They then proposed questions that could potentially uncover these risk factors when posed to users and suggested some guidance on what HCPs should consider during a consultation. In the second round, the boards reviewed the outcome of the previous sessions and refined the existing questions and the guidance information. Finally, in the third round, the boards evaluated the tool by using it in a simulation.

### Development of the tool

The proposed questions were merged and organised into a logical flow and categories together with appropriate guidance and recommended actions based on the nurses’ input and literature findings. The questions and content were also rephrased from a user’s perspective.

The format was developed by employing a variety of methods according to the steps of the Design Thinking Process, including creation of personas and scenarios, as well as prototyping (see Fig. 1). A prototype of the physical tool was tested in the third round of Nurse Advisory Boards’ meetings, where nurses used the tool in a simulation (role play). The nurses subsequently provided feedback on the content, language, and format, which was then incorporated in the final version of the tool.

## Results

### Qualitative evidence synthesis

**Fig. 2:**
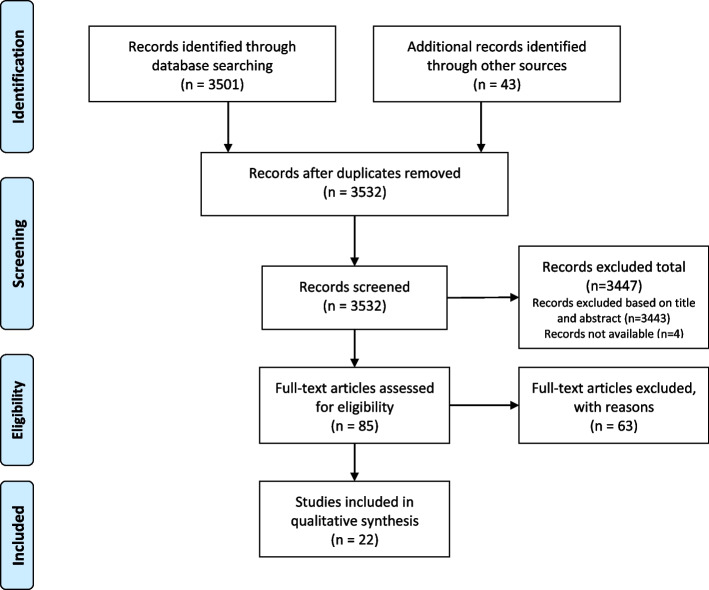
PRISMA Flow chart

The database search identified 3501 results. After removing duplicates and screening titles and abstracts, 81 articles were identified, out of which 17 met the inclusion criteria. One case report article was also included. Additional records were identified based on the references of included articles, out of which four articles were included in the findings (Fig. 2).

The analysis was based on the research question “how do intermittent catheter users experience having a UTI and which factors do they describe influences recurrent UTIs?” The themes that emerged from the literature were related to the relationship between UTIs and health, adherence, technique, and catheter choice. These findings are described below.

#### Effect of UTIs on health

UTIs affect the overall health and energy, they require treatment and may lead to hospitalisation [22, 25]. Additionally, frequent UTIs can impact the work life, social life, and intimate relationships, eventually leading to social isolation and low user satisfaction [26–28]. However, the perception of UTIs varies among individuals from something inherent to IC to a cause of relapses of Multiple Sclerosis [26]. Self-monitoring for UTIs can be empowering, giving a feeling of control over maintaining one’s own health, while other users experience anxiety over the uncertainty of the timing and severity of the infection [22, 29].

With time, users start understanding their body and recognising UTI symptoms and the need for treatment [26, 30]. However, it may be difficult to distinguish UTI symptoms from those related to comorbidities or age. Notably, users may describe their symptoms using a language that does not match the clinical terms. Therefore, the lack of a user-friendly list of signs and symptoms may hinder UTI diagnosis and management [18].

#### Impact of non-adherence on UTIs

Lack of adherence is associated with increased risk of UTIs [12]. Adherence is not only related to knowledge and the ability to perform IC, but also to feelings and fears [31]. Users’ decisions to adhere to IC can be motivated by “*psychosocial desires”*, such as a need to avoid embarrassing incontinence episodes and to feel confident [22]. For some users, IC is a reminder of the impairment and thus, resisting adherence is “*an act of resisting abnormality*” [31]. This is an act with immediate positive psychological effects, but long-term complications [29]. Challenges and changes in the IC technique, anatomical barriers, complications such as pain, bleeding, and discomfort, as well as the dependence on assistance, obstruct the recommended IC frequency [32–35]. Fears of infection, pain and self-harm and the struggle to adapt new routines into those practiced for years also interfere with adherence [31]. Adherence may also be challenged when UTIs occur early in the life with IC, as users may doubt the effectiveness of this method [21]. Finally, performing IC away from home is troublesome. It may require catheterisation from a different position [32, 33], while public toilets are seen as a cause of UTI due to lack of sanitary, accessible bathrooms [7, 18, 22, 32, 34, 36].

IC users develop self-help methods and strategies that give them control over their everyday life, and promote feelings of normalisation and independence [29]. Routines, rituals and workaround strategies help maintain a regular catheterisation schedule [22, 36]. This schedule, along with a practice of increasing fluid intake after observing UTI symptoms were viewed as preventive strategies against UTIs [18]. However, some strategies can also adversely impact the risk of UTIs. For example, users may reduce fluid intake during a day away from home or when they are in the hospital to avoid the need for toileting assistance from the staff [22, 30, 37].

#### Impact of technique and education on UTIs

Some users have physical barriers to handling the catheter properly, for example, reduced dexterity or spasticity [7, 21, 37, 38]. The technique can also be challenging for female users because the urethra is less accessible [7, 28, 34, 38]. However, education on IC is also often insufficient, predisposing users to incorrect technique and misconceptions around the benefits, functionality, and complications of IC [31, 39]. Lack of knowledge was particularly noted in terms of anatomy, disease, symptoms, and aetiology of UTIs, while an array of negative feelings including anxiety, fear, shock, depression, and stigma were associated with the IC training [31, 38–41].

As a result, users may leave the training session uncertain of the technique and even learn about their anatomy by trial and error [30, 35]. They are nonetheless hesitant to contact HCPs with questions, indicating that long-term IC users may not have received up-to-date education on best practices [18, 29]. Users valued follow up care as it could help them maintain the correct technique and felt that information on hygiene and UTI prevention would help them avoid complications [35, 41].

#### Impact of catheters on UTIs

Access to catheters and the opportunity to try different products varies from country to country [29, 30, 32]. The potential for re-using catheters, which is dependent on the user’s country of residence, was primarily associated with worry about UTIs, as the single-use method was viewed as cleaner and more sterile [42].

When given a choice, different preferences on the catheter features were mentioned. For example, users talked about choosing between a narrower diameter to minimise discomfort or a wider diameter that reduced voiding time, while the length affected whether the bladder could be fully drained and if the catheter could reach the toilet. Preferences concerning the rigidity of the catheter were balanced between control of navigation and damage to the urethra, whereas lubrication influenced the ease of insertion and handling [33]. When away from home, using an alternative catheter such as a pre-lubricated one was mentioned [33]. Overall, trust in the product design and quality was highlighted and catheter properties that reduced discomfort and the perceived risk of trauma were favoured [25, 33].

### Nurse advisory board input

Advisory Board nurses found that the most relevant UTI risk factors to assess were related to the user’s general health condition, their adherence, technique, and the catheter type used. They highlighted that before evaluating risk factors, they would confirm whether the person has a UTI that requires treatment. The questions the nurses proposed were subsequently organised into groups assessing the following: UTI confirmation, risk factors related to health, adherence, technique, and catheter and, finally, supporting questions to conclude the consultation. The findings from the Advisory Board meetings are outlined below.

#### UTI confirmation

Before initiating treatment, nurses agreed on the importance of confirming whether the user has a UTI, based on the guidelines, to avoid the risk of overtreatment [10, 43]. Therefore, diagnostic tests could be supplemented by questions on experienced symptoms and how users recognise a UTI.

Important insights can be collected during this step, that could guide the risk factor assessment. These concern the history of UTIs, including recurrency of UTIs and antibiotic resistance, as well as patterns and triggers of UTIs that users may have already identified or suspect. HCPs should also understand the user’s challenges, as well as their fears, and the psychological impact UTIs have on the user’s life.

#### Health

An overall deterioration in the user’s health condition was recognised as a potential reason behind UTIs. Furthermore, ageing and menopause may affect the urinary tract and lead to more UTIs and symptoms. With the user’s specific diagnosis and demographics as a starting point, the Advisory Boards recommended exploring health changes or medication that may influence the bladder and bowel, such as medication for diabetes, high bladder pressure and, naturally, bladder and bowel medication. Furthermore, bowel function should be examined in terms of frequency and stool type, as should incontinence or constipation problems, which increase the risk of UTI. The bowel situation can be mapped by using the Neurogenic Bowel Dysfunction (NBD) score [44], the Bristol stool scale [45] and the MENTOR tool [46]. Additionally, the wipe direction should be assessed, especially in persons with limited dexterity.

#### Adherence

The discussion also centred around the importance of users acknowledging the benefits of IC and how adherence to the IC protocol prevents UTIs. It was recommended to ask the user how IC fits into their life, discuss opportunities for the future, explore their practices for maintaining a healthy bladder and try to understand underlying beliefs or misconceptions about IC and UTIs. When experiencing complications like soreness or a UTI, it is critical to solve these problems, while emphasising the importance of not switching to another method or reducing the catheterisation frequency.

The 3-day bladder diary was considered an insightful method used both by urologists and nurses to adjust the catheterisation frequency and fluid intake [47]. For example, low water consumption may be observed during specific time points, while incontinence episodes may indicate that the recommended bladder capacity is exceeded between catheterisations. The Advisory Boards recognised that the diary is cumbersome for users but acknowledged that even an incomplete diary can provide insights. However, if a diary is completely unavailable, the HCP can instead ask about factors such as fluid intake, urine output, IC frequency and the colour of the urine. For users who void spontaneously in addition to performing IC, the total volume of urine output should be considered.

#### IC technique

The Advisory Boards noted that the IC process varies between users, depending on, among other possibilities, sex, catheter type (e.g., single or re-use), the recommended technique (no-touch or clean) and the catheterisation position (sitting on toilet, in a wheelchair or lying down). It is therefore important to assess all the steps from preparation to insertion and withdrawal of the catheter. Along with asking questions, they emphasised that observing the user perform IC can reveal discrepancies between understanding and practice and can also highlight unspoken issues that could otherwise remain unnoticed.

Following the complete catheterisation process, attention points start with hygiene practices related to cleaning hands and the genital area, and the hygienic handling of the catheter when opening the package and preparing it for use. The lubrication step should be observed, if relevant. It is also important to take note of whether the catheter touches surfaces, the clothes, or the body, if the meatus becomes well visible and if the catheter is inserted far enough. Other important aspects to observe include the positioning of the catheter, and whether complete bladder emptying is ensured, for example by withdrawing the catheter in small steps, or repositioning the catheter or the body.

Reviewing these steps allows the HCP to assess if the technique recommended to the user is followed correctly and if an adjustment is needed to reduce the risk of UTI. Prior to correcting the technique, asking users about their perceived difficulties with IC empowers them to reflect and take more control in determining the steps they could improve with their technique.

#### Catheter choice

When selecting a catheter, the nurses remarked that it should fit into the user’s personal situation and preferences, while different types of catheters may be preferred for different situations.

The type and size of the catheter are among the most important determinants of functionality. Examples mentioned included catheters requiring lubrication or activation by the users which must be properly prepared, while catheters that dry out quickly may not be suitable for users that require a longer time to catheterise. Symptoms such as pain, discomfort and bleeding could be indicators that the catheter coating or design are not optimal for the user.

#### Support for users

Follow up support is essential to ensure users are comfortable with the technique and are up to date with best practices. It was therefore proposed that users be asked what information and support they would need to better handle and prevent UTIs. HCPs could thus ensure users receive the correct information on UTIs and could have an opportunity to offer other relevant health or lifestyle advice.

#### Tool format

The Nurse Advisory Boards highlighted that the questions in the tool should be phrased in such a way that they do not worry the user but, at the same time, succeed in obtaining relevant information. Questions should therefore be open-ended yet straightforward. It is also crucial that HCPs have background knowledge about the tool’s questions so that they can explain why obtaining this information is important.

**Fig. 3:**
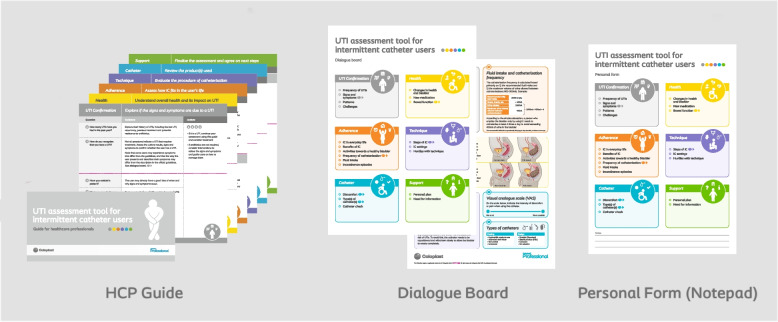
The three elements of the UTI assessment tool for intermittent catheter users: A Guide for healthcare professionals, a Dialogue board, and a Personal form notepad

**Fig. 4:**
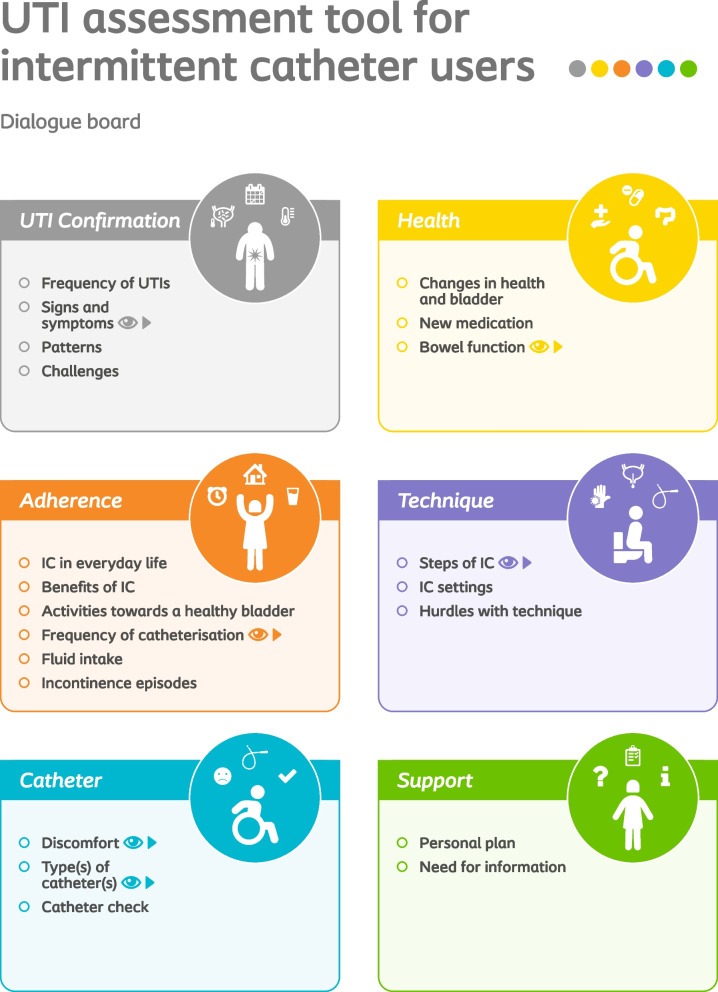
The six sections and the topics included in the UTI assessment tool for intermittent catheter users, as illustrated in the Dialogue board

Based on the collected input, the designed tool consists of three elements (Fig. 3):

1. The HCP guide, which contains a structured list of questions, organised in six sections: UTI Confirmation, Health, Adherence, Technique, Catheter and Support. Each question is accompanied by guidance text, that can support the HCP, as well as relevant follow-up actions.
2. A Dialogue Board (Fig. 4) intended to engage the user. The front side is divided into the same six sections as above and contains headers that correspond to each question. The back side of the board features illustrations and explanations that include an adapted list of UTI signs and symptoms and a VAS scale can help users describe their symptoms. Anatomic illustrations visualising the bowel’s influence on the bladder and the need to reposition to avoid residual urine are also included to support users’ understanding and training. A simple example outlines the calculation of the recommended catheterisation frequency and, finally, a list of available catheter designs can be a way to include the user in the selection of an appropriate product. Overall, the dialogue board supports the HCP in explaining some concepts to the user, while also helping the user to follow the conversation, understand the relevance of UTI risk factors and take an active role in their management.
3. A personal form containing sheets featuring the dialogue board with space for note taking can be used to record information relevant to the individual user which they can take home.

## Discussion

During this study we collected input using a two-step approach, via a qualitative evidence synthesis and discussions with nurses that helped develop questions that can uncover UTI risk factors.

In the literature we found that UTIs caused anxiety due to the uncertainty around their occurrence and that it took time for users to learn how to recognise UTI symptoms [22, 26, 29, 30]. Some users found it difficult to report the symptoms experienced, which could lead to misunderstandings [18]. Uncertainty about risk factors, lack of up-to-date knowledge and misconceptions about IC and UTIs were noted and did affect adherence [31, 39]. Users talked about the impact that UTIs and IC have on their lives and expressed a diversity of views on these topics. The literature abounds with contradictory points of view, which are not to be interpreted as data irregularities, but rather as testimonies that it is not only the physiology, but also - and even more importantly - the psychosynthesis of users that is different. This emphasises the importance of obtaining a holistic understanding of the problem, exploring the user perspective and, ultimately, identifying individualised risk factors and solutions.

In the HCP perspective, as expressed by the Advisory Boards, we found the same themes (overall health, adherence, technique, and catheter), but addressed with a clinical focus. The nurses advised to explore each theme with straight forward and open-ended questions and stressed that it was important for HCPs to have solid background knowledge, to be able to explain the importance of these topics in preventing UTIs to the user.

The UTI assessment tool for intermittent catheter users was developed by combining the findings from the literature and the Nurse Advisory Boards. One of the challenges identified was the uncertainty about signs and symptoms of UTIs among IC users, along with the confounding effect of symptoms occurring from other conditions [18]. This includes situations such as impaired psychological state and social well-being [48] or physiological problems such as bowel dysfunction. Furthermore, some users may have reduced sensitivity secondary to the underlying neurological disease, and present atypical symptoms such as increased spasticity [49]. These uncertainties, which are only aggravated by the inconsistency in UTI definitions [43, 50], highlight the need for HCPs to confirm the UTI cases and avoid antibiotic treatment of asymptomatic bacteriuria, a problem that despite the high level of evidence still needs attention [51, 52]. An increased education of HCPs and users is also required to identify symptoms and risk factors and effectively prevent UTIs.

We believe that we have created a tool that allows HCPs to identify and address UTI risk factors in a systematic way, while putting the user’s perspective in focus. To our knowledge this is the first tool that includes users’ perspectives in its development. It provides a structured dialogue approach to preclude missing important aspects of UTI assessment, while being adaptable to the individual user. A user-centric approach is maintained in the way that questions are phrased and organised, while the open-ended formulation of the questions is designed to grasp and address the user’s understanding and potential misconceptions. Furthermore, general questions and inquiries about the user’s life are placed in the beginning of the assessment to build a sense of ease and overcome privacy barriers, before moving to a demonstration of the catheterisation process. Finally, this tool concludes by building a contract with the user about the next steps and ensuring that their education is accurate and relevant.

The development of the tool has been conducted in a structured way that considers both user and HCP perspectives. It is, however, acknowledged that input was only collected from nurses within Europe, where the standard practice is the no-touch technique with single-use hydrophilic coated catheters. Therefore, in its implementation, some aspects of the tool may need to be adapted to the local culture and best practices.

## Conclusion

The aim of this study was to develop a tool to help HCPs evaluate the UTI risk factors for individual users in a systematic way and identify areas of focus for interventions. By including the perspectives of experienced nurses and users, as described in the literature, we have succeeded in developing the UTI assessment tool for intermittent catheter users. This tool will help assess UTI risk factors that are relevant for individual users in a systematic way and via a holistic approach that considers the overall health, adherence, technique, and catheter use, while also considering the user perspective. Subsequently, the tool will allow HCPs to focus interventions on the relevant causes of UTI, thus improving the quality of the assessment and management of UTIs.

The tool will be introduced in a range of international and national conferences and events, as well as in seminars driven by Coloplast. Observation of the tool in real-life settings should allow the validation of its intended function and its influence on preventing UTIs. Finally, it is acknowledged that this tool is only an aid and can never exceed the HCP’s own experience and curiosity to understand and support the users in their care.

By request the UTI assessment tool for intermittent catheter users can be obtained in local language, if available, by contacting the local Coloplast office.

## Data Availability

The datasets (quotes) analysed during the current study are available from the corresponding author on reasonable request.
